# An Active Beam‐Reconfigurable Retrodirective Array

**DOI:** 10.1002/nap2.70200

**Published:** 2026-07-04

**Authors:** Wen Yue Wei, Yan Shi, Li Jing Yang, Jun Wei Wu, Qiang Feng, Long Li

**Affiliations:** ^1^ School of Electronic Engineering Xidian University Xi'an China; ^2^ State Key Laboratory of Millimeter Waves Southeast University Nanjing China

**Keywords:** active amplifiers, dual‐beam, reconfigurable array, reconfigurable steering beam, retroreflection

## Abstract

Retrodirective arrays can scatter an arbitrarily incident wave back along its incident direction, demonstrating significant potential for wireless communications. However, traditional retrodirective arrays can only reflect incident waves back along a fixed direction. This inherent constraint restricts their application primarily to point‐to‐point communications. To overcome this constraint, this paper focuses on the study of a beam‐reconfigurable retrodirective array. The proposed array consists of dual‐beam radiating elements. By incorporating a metallic plate with a tunable tilt angle, the array not only achieves retroreflection but also generates a reconfigurable steering beam. Furthermore, integrating active amplifiers into the array's transmission line network enhances the gain of both beam types. The proposed array system not only responds in real‐time to incident waves from different directions but also enables dynamic switching among various steering beams. This dual capability opens up diverse application potentials, including multipoint wireless communication and multitarget wireless power transfer.

## Graphical abstract

The proposed beam‐reconfigurable retrodirective array not only enables retroreflection toward the signal source but also achieves flexible beam steering via a tunable reflection plate, offering a viable solution for real‐time multipoint communication and multitarget wireless power transfer.

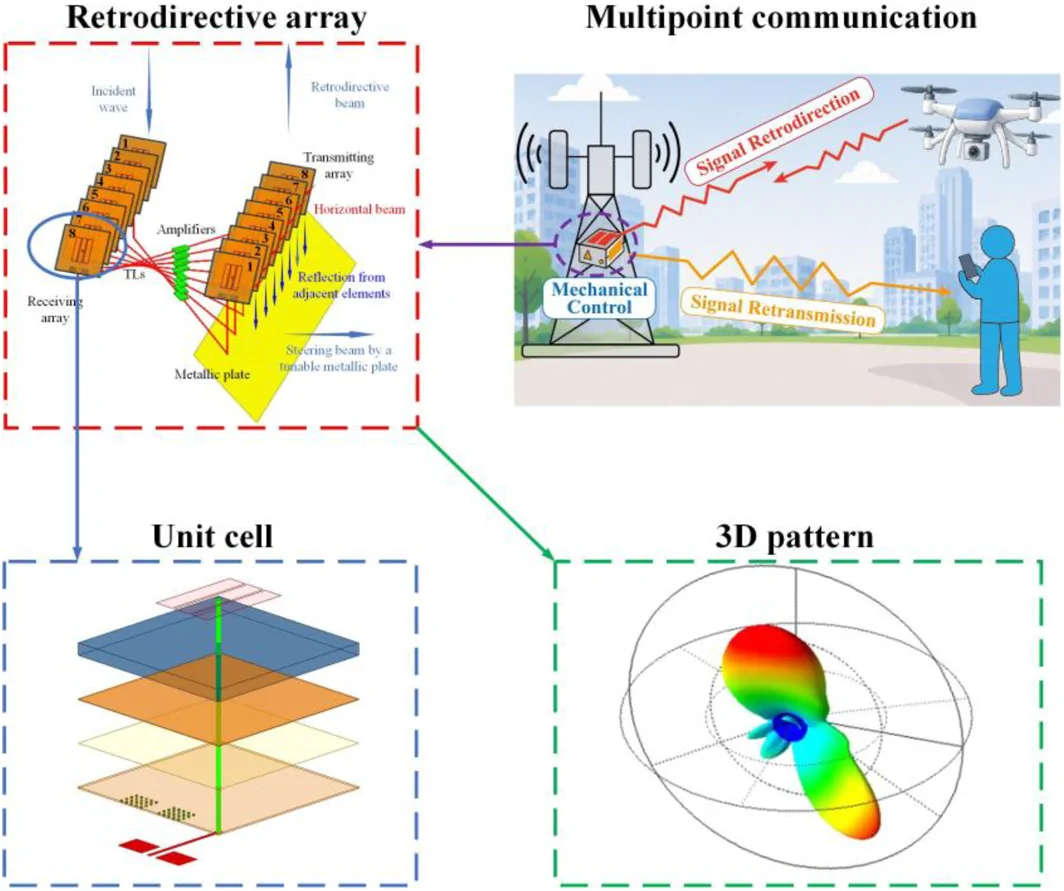

## 1 Introduction

The rapid development of metamaterials over the past few decades has been driven by their extraordinary ability to manipulate electromagnetic (EM) waves. Composed of periodic or quasiperiodic microstructured elements, metamaterials exhibit remarkable physical properties in specific frequency bands, overcoming the limitations of natural materials. However, traditional three‐dimensional metamaterials face practical constraints due to their bulky configurations. With the progressive thinning of structural profiles, metasurfaces have emerged as the two‐dimensional equivalents of volumetric metamaterials, enabling effective control over the amplitude, phase, and polarization of EM waves. Metasurfaces offer a compelling alternative to bulky metamaterial architectures, with notable advantages such as reduced footprint and lower losses. As a result, they have attracted significant interest from researchers worldwide, with a growing body of literature exploring diverse applications. Numerous studies have been dedicated to exploring the diverse applications of metasurfaces, particularly in scattering‐field manipulation [1, 2, 3, 4] and beamforming [5, 6, 7, 8, 9, 10, 11, 12, 13, 14, 15, 16, 17, 18, 19]. Such metasurfaces are capable of steering beams toward predefined directions in real time. However, when communicating with moving objects, they fail to maintain an effective communication link, primarily due to the lack of critical beam retrodirection capability and the consequent inability to locate moving targets.

Retrodirective arrays have emerged as effective self‐beam‐steering devices, capable of reflecting incident waves back toward their original direction regardless of arrival angle. This characteristic enables their widespread application across multiple fields, including wireless communications, RFID systems, and electronic countermeasures. Conventional implementations of retroreflection include corner reflectors, Luneburg lenses, and cat's eye retroreflectors [20, 21, 22, 23, 24, 25]. However, their considerable size and nonplanar geometry pose significant integration challenges with other components, thus restricting their practical deployment. As a planar alternative, Van Atta array achieves adaptive retrodirection through a specific array arrangement and feeding network, thus providing a low‐profile solution with enhanced integration compatibility [26, 27, 28, 29, 30, 31, 32, 33, 34, 35, 36, 37, 38, 39]. A conventional Van Atta array consists of multiple radiation elements connected in pairs via transmission lines of equal electrical length. This configuration inverts the phase progression of the incident wave, thereby enabling automatic retroreflection. Despite this advantage, the array produces a reflected beam solely toward the source direction once fabricated, lacking any capability for beam steering or modulation. Moreover, as a passive structure, its relatively low radar cross‐section (RCS) further restricts its applicability in wireless communication.

In recent years, the emergence of metasurface technology has prompted researchers to explore new approaches for achieving retrodirective functionality [40, 41, 42, 43]. For instance, a hexagonal metasurface‐based electromagnetic grating has been proposed, capable of efficiently retro‐reflecting signals incident from six distinct directions over wide angles, with a reflection efficiency exceeding 85%. However, such metasurface arrays share similar functionalities and limitations with conventional retrodirective structures—once fabricated, their reflection behavior becomes fixed, lacking the ability to dynamically control the reflected beam [44]. To address this limitation, a reconfigurable active Van Atta array has been introduced, incorporating PIN diodes into the transmission line network to enable electrical control over the reflection state. When the diodes are switched on, the array maintains its retrodirective function; when turned off, the interelement connections are disrupted, causing the array to behave as a metallic plate that produces specular reflection. However, the reflected beam direction is fixed for a given incident wave. Consequently, this limited adaptability hinders its practical deployment in complex electromagnetic environments [45]. In addition, an intelligent reconfigurable metasurface system has been proposed to realize adaptive functions such as retrodirection, directional reflection, and beam focusing. Moreover, dynamic multibeam manipulation can also be realized by controlling the reflection phase profile of metasurface unit cell. However, achieving retroreflection toward an unknown incoming direction necessitates direction‐of‐arrival (DOA) estimation, which inevitably increases system latency and reduces response speed [46].

This paper presents a beam‐reconfigurable retrodirective array (BRRA). A dual‐beam radiating element is first designed, serving as the building block for the retrodirective array. Multiple power amplifiers (PAs) are integrated into the transmission line (TL) network to enhance the RCS of the array, enabling a longer operational range. Furthermore, a dynamically controllable metallic backplane is introduced beneath the transmitting array. This enables the system not only to perform retrodirection toward the incident wave, but also to generate an additional scattering beam with a steerable direction. A prototype was fabricated and experimentally characterized, validating the performance of the proposed BRRA. The dual‐beam capability significantly expands the applicability of retrodirective arrays, moving beyond traditional point‐to‐point retrodirection to include point‐to‐multipoint applications, such as multipoint wireless communication or multiobject wireless power transfer. Figure 1 depicts a street communication scenario with a moving unmanned aerial vehicle (UAV) and a walking pedestrian. To maintain reliable dual‐link connectivity from the base station to both the UAV and the pedestrian, the proposed BRRA, mounted on the base station tower, generates two independent beams: a primary retrodirective beam that automatically tracks the UAV without angle‐of‐arrival estimation, and a secondary beam whose direction is dynamically steered to follow the pedestrian.

**FIGURE 1:**
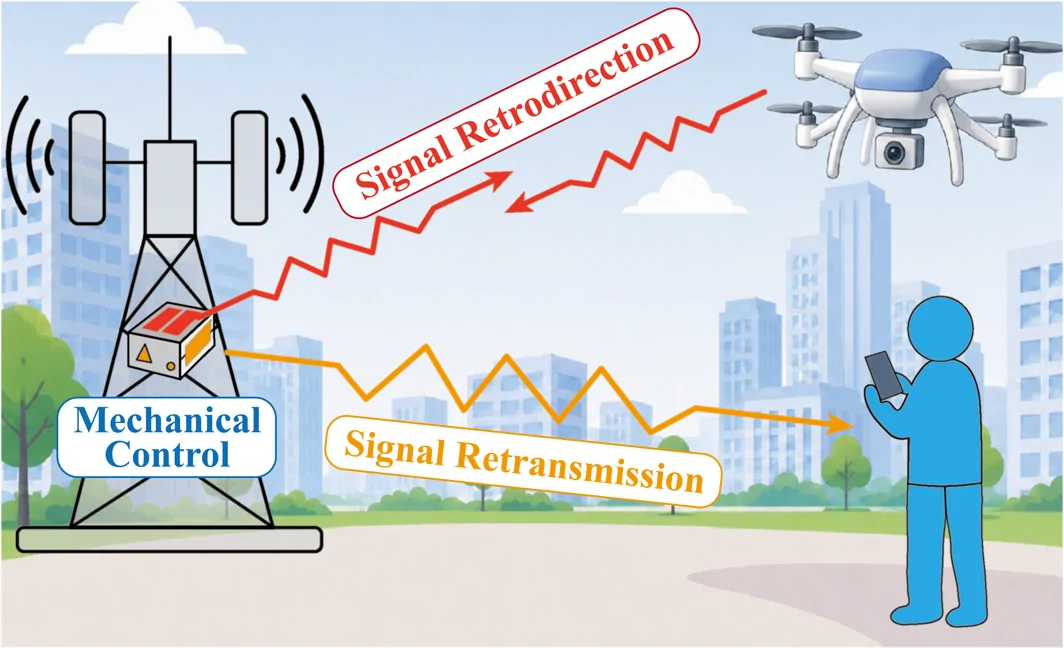
Schematic of the proposed BRRA for point‐to‐multipoint communication including moving targets.

## 2 Results

### 2.1 Methods and Materials

To validate the proposed BRRA, a 16‐element array system is designed. The system primarily consists of a retrodirective array integrated with an active power‐amplified TL network and a dynamically controllable metal backplane. The design procedure of the retrodirective array system is outlined as follows:

1. Designing the structure and dimensions of the element to ensure desirable dual‐beam radiation characteristics within the operating frequency band.
2. Designing power amplification modules to enable active enhancement of the array's RCS.
3. Integrating the power amplification modules into the TL network and connecting the active TL network to the elements to achieve a complete array system.
4. Placing a flexibly controllable metal backplane beneath the retrodirective array and adjusting its configuration to achieve dynamic control over an additional steering beam, apart from the retroreflection beam.

#### 2.1.1 Radiating Element Design

As illustrated in Figure 2a, a multilayer structure is designed as a dual‐beam microstrip element operating in the X‐band, which is centrally fed by a coaxial line to excite the radiating patch. The top dielectric substrate employs F4B material with a relative permittivity of 3.5 and a thickness of 2 mm. As depicted in Figure 2b, a rectangular patch fabricated on the top surface of F4B substrate functions as the receiving/radiating element. Two slots along the *x*‐direction are introduced to guide the surface current, thereby enabling dual‐beam radiation. The middle layer is a metal ground plane, featuring a circular aperture to allow the passage of the feeding pin. The bottom substrate is made of F4BTMS1000 with a permittivity of 10.2 and a thickness of 0.254 mm. A microstrip feedline, comprising a circular pad and a linear section, is fabricated on the bottom surface of F4BTMS1000 material. The circular pad is connected to the feeding pin to ensure effective signal transmission. Due to the presence of the intermediate ground plane, an SMP soldering pad is integrated at the feedline input, and grounding vias are incorporated to extend the ground reference to the feedline layer, facilitating the soldering of RF connectors. The two dielectric layers are bonded using Rogers 4450 prepreg with a permittivity of 3.5 and a thickness of 0.1 mm, enabling a low‐profile configuration. Detailed geometric parameters of the radiating element are summarized in Table 1.

**FIGURE 2:**
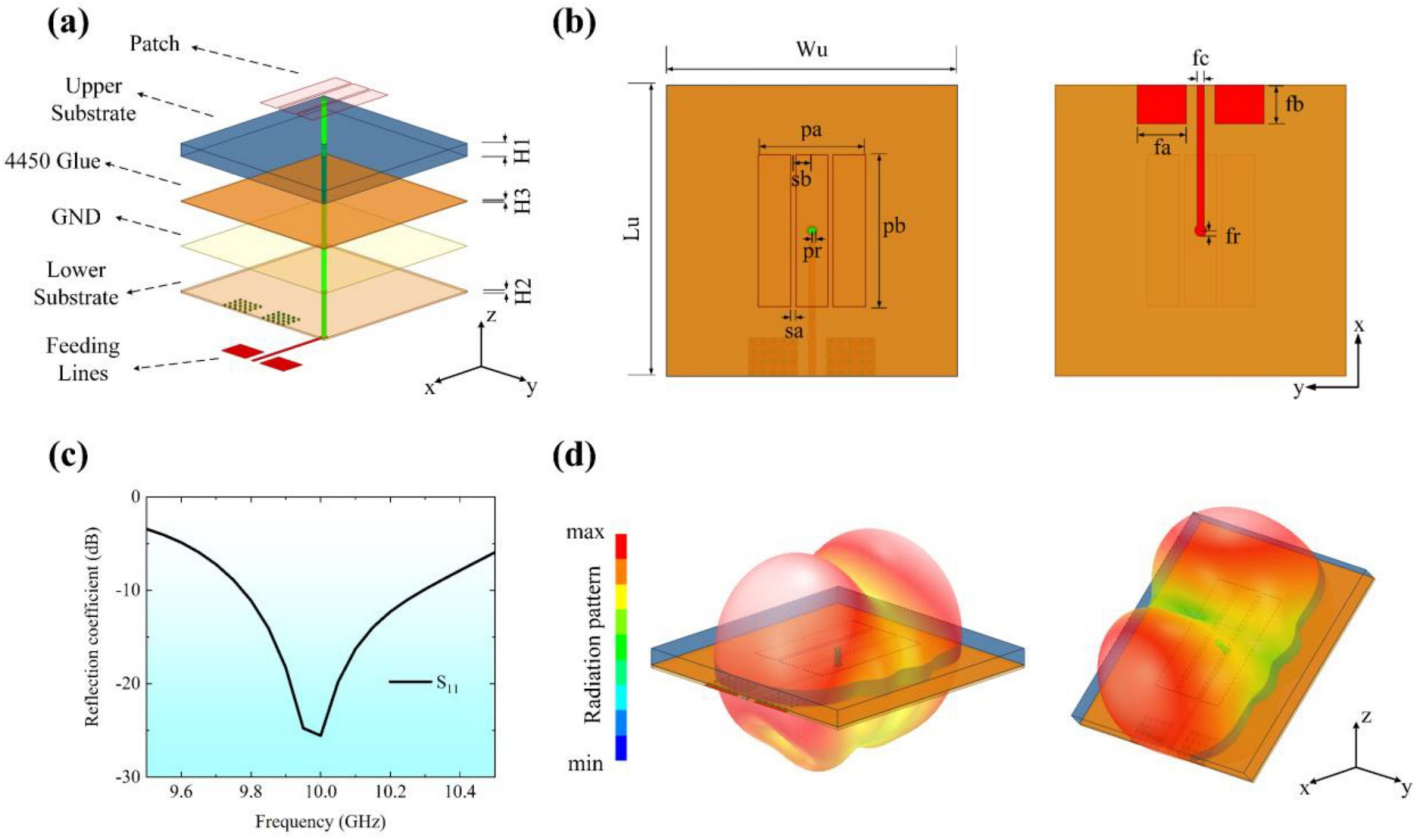
(a) Schematic diagram of radiating element structure. (b) Dimensioning of the element. (c) Reflection coefficient of the element. (d) 3D radiation pattern of the element.

**TABLE 1:** Detailed size of the radiating element.

Parameters	Value [mm]
Lu	30
Wu	30
Sa	0.5
Pa	11
Pb	15.7
Sb	1.9
Fa	5
Fb	4
Pr	0.4
Fc	0.65
Fr	0.6

The performance of the element was analyzed using the commercial full‐wave electromagnetic simulator Ansys HFSS. As shown in Figure 2c, the simulated reflection coefficient remains below −10 dB over the frequency range of 9.78–10.29 GHz, with resonance occurring at 10 GHz. Furthermore, Figure 2d presents the radiation pattern of the proposed element, exhibiting a dual‐beam profile symmetric about the broadside direction. Specifically, the two beams reach their maximum gain of 7.15 dBi at approximately *θ* = 45° and *θ* = −45°, respectively, with nearly identical 3 dB beamwidths of 57.62° and 57.8°. When the unit cell is rotated 45° about the *y*‐axis, the two beams shift to the *θ* = 0° and *θ* = 90° directions in the *xoz* plane. The first beam at *θ* = 0° serves for retroreflection, whereas the second beam at *θ* = 90° is designed to function as a tunable steering beam.

#### 2.1.2 TL System and Active Amplification Module

To enhance the intensity of two beams in the retrodirective array for long‐distance communication, eight PAs are embedded into the TL network as an active amplification module. The QPA‐2610 X‐band PA from Qorvo is selected, which operates across 8.5–10.5 GHz, provides a gain of up to 37.5 dB within the band, and delivers a maximum output power of 33 dBm (2 W). The static operating points are set as *I*_*D*_ = 56 mA and *V*_*D*_ = 20 V. The device is packaged in a compact 5 × 5 × 0.85 mm form factor. Figure 3a illustrates the physical configuration of the PA module. An aluminum alloy block is used at the bottom to improve heat dissipation, whereas the upper part is enclosed by a 3D‐printed housing covered with copper tape for electromagnetic shielding, thereby mitigating mutual interference among amplifiers and other RF components. Mounted on top of the heat sink are the amplifier chip board and the bias circuit board. The chip board is fabricated on a Rogers RO6010 substrate with a relative permittivity of 10.2 and a thickness of 0.635 mm, with a silver‐colored chemical silver finish serving as the surface treatment. The chip is positioned at the center of the board, with solderless SMA RF connectors on both sides for signal transmission. The bias circuit board, green in color, is fabricated on an F4B substrate with a relative permittivity of 3.5 and a thickness of 2 mm and is responsible for supplying power to the amplifier.

**FIGURE 3:**
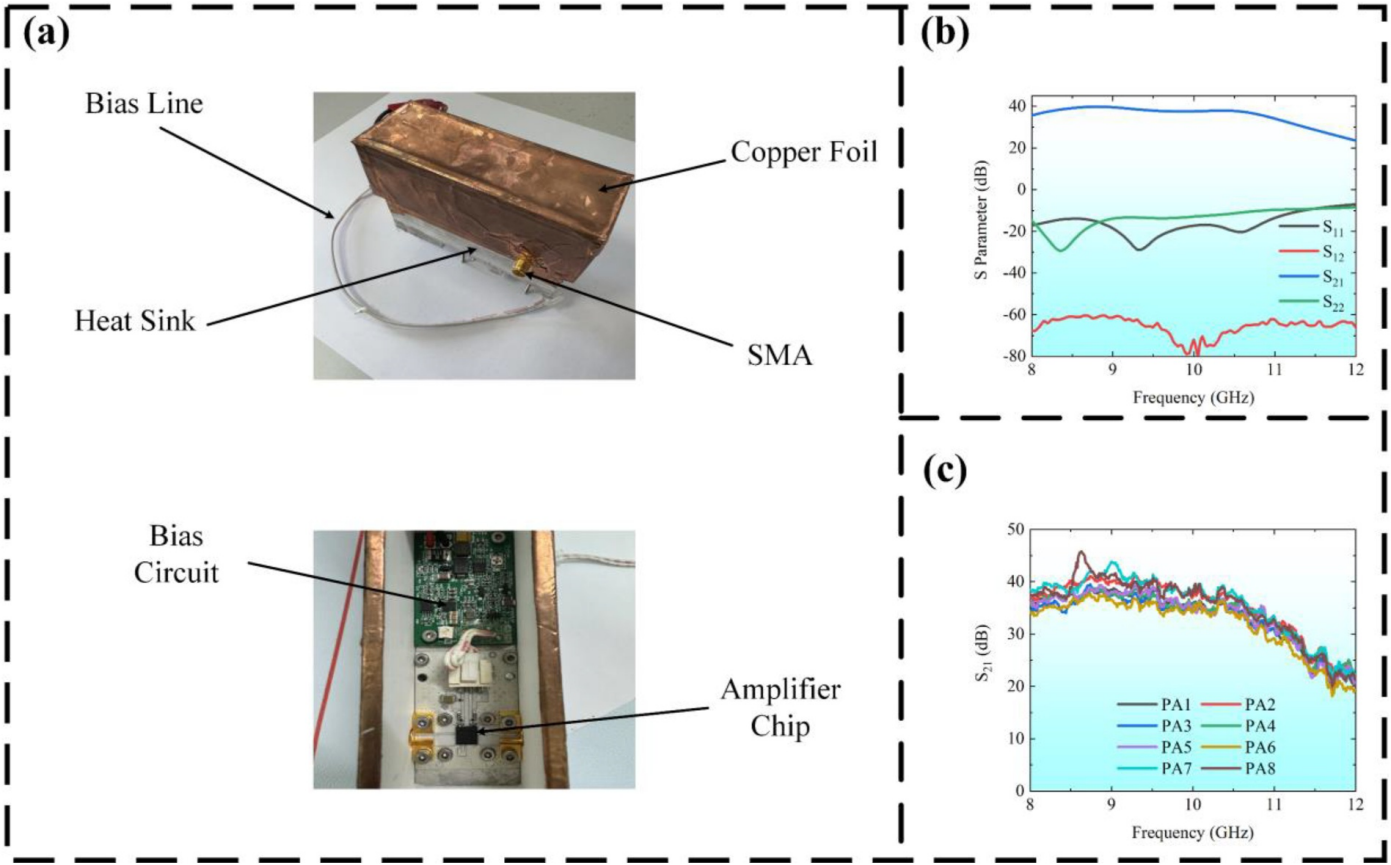
(a) Physical photograph of power amplifier. (b) Simulated S‐parameters of the power amplifier. (c) Measured S_21_ of the 8 power amplifiers.

In the power supply design, *V*_*G*_ and *V*_*D*_ are connected to a negative voltage and a positive voltage, respectively. The power‐on sequence follows “negative before positive,” whereas the power‐off sequence is “positive before negative.” The VG line is equipped with three capacitors (10 μF, 0.1 μF, and 1 nF) and two resistors (0 and 10 Ω). The VD line includes two capacitors (0.1 μF and 1 nF) and one resistor (10 Ω). The 10 μF capacitor is used to adjust the power‐on timing, whereas the small‐value capacitors serve to filter high‐frequency noise. The resistors contribute to improved filtering and circuit protection. A total of eight PA modules were fabricated and assembled for subsequent integration into the array.

The performance of the PA is simulated in CST Studio Suite to analyze its S‐parameters over the X‐band. The results, presented in Figure 3b, show that both the input and output ports are well matched across 8–11 GHz, with S_11_ and S_22_ below −10 dB. Moreover, the reverse isolation S_12_ remains below −60 dB throughout the band, whereas the gain S_21_ exceeds 30 dB across the entire frequency range, reaching 37.62 dB at 10 GHz.

Under the bias conditions of *V*_*G*_ = −2.8 V and *V*_*D*_ = +23.8 V, the transmission coefficients of the eight PAs were measured using a vector network analyzer. It can be observed from Figure 3c that all eight amplifiers exhibit similar gain trends, which align well with the simulation results. Minor variations in the absolute gain values are mainly attributed to inconsistencies introduced during the soldering process.

#### 2.1.3 Retrodirective Array System and Simulation Results

As illustrated in Figure 4, the whole retrodirective array consists of two separate subarrays. The left subarray serves as the receiving array, whereas the right one acts as the transmitting array. Notably, instead of using a single integrated array, two separate subarrays are employed to ensure sufficiently high isolation between reception and transmission, thereby avoiding self‐oscillation of the power amplifiers. Both subarrays comprise eight elements with an interelement spacing of 16 mm (0.53λ at 10 GHz) to ensure adequate scanning performance of the array pattern. Each element is rotated 45° about the *y*‐axis to radiate toward 0° and 90° directions. The two subarrays are parallel arranged with a center‐to‐center distance of 50 mm. In order to achieve the retroreflection, a group of TLs are introduced to connect the receiving array with the transmitting array. Specifically, the element *i* in the receiving array is connected to the element *i* in the transmitting array (*i* = 1, 2,…, 8). An amplifier is integrated into each TL to amplify the signal.

**FIGURE 4:**
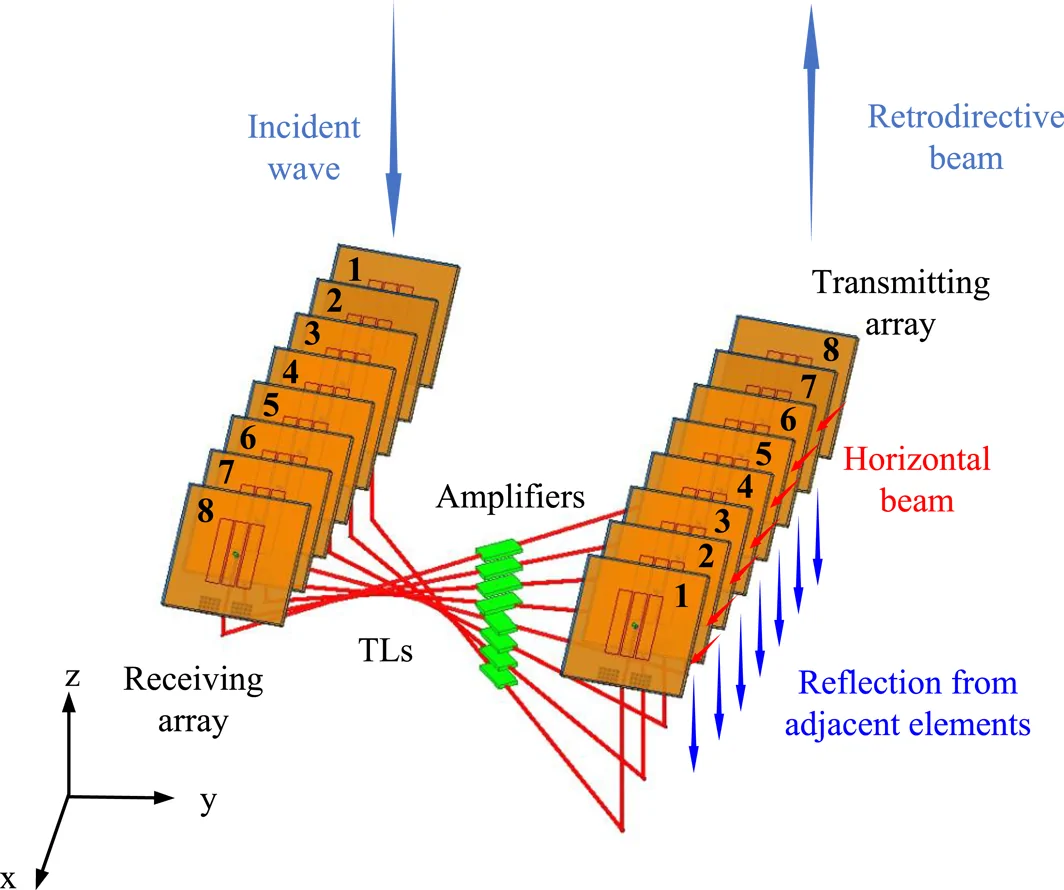
Dual‐beam retrodirective array system including a transmitting subarray, a receiving subarray, and a TL network integrated with amplifier modules.

When an *x*‐polarized incident wave normally illuminates the array along ‐*z* axis, the 0°‐direction beam of receiving elements capture the energy, which is then reradiated by their symmetrical counterparts in the transmitting array. The 0° direction beam of the transmitting element forms the retrodirective field, whereas the 90° direction beam of the transmitting element impinges on the metal backplane of adjacent elements in the transmitting array and thus is deflected toward –*z* direction by specular reflection. When an electromagnetic wave impinges on the receiving array at an oblique angle *θᵢ*, the captured signal is routed through amplifier‐loaded TLs to the transmitting array, which then radiates it in two directions. Thanks to the centrally symmetric TL network linking the receive and transmit arrays, the system simultaneously generates a retroreflective beam along *θᵢ* direction and a complementary beam along 180°−*θᵢ* direction.

The proposed dual‐beam retrodirective array is analyzed using ANSYS HFSS. A *θ*‐polarized incident wave operating at 10 GHz illuminates the retrodirective array in the *φ*_*i*_ = 0° plane (i.e., the *xoz* plane), with the incident angle *θ*_*i*_ undergoing a sweep from −30° to +30°, which corresponds to the maximum range limited by the radiating element's beamwidth. As shown in Figure 5a, the array generates two distinct scattering beams: a well‐defined retroreflective beam covering from −30° to +30°, and a steerable beam spanning from 150° to 210°. For example, with an incident angle *θᵢ* = 30°, the array produces a retroreflective beam at *θ* = 30° and a steering beam at *θ* = 150°, consistent with the predicted beam pattern. Figure 5b also presents the 3D scattering field patterns for incident waves at *θ*_*i*_ = 30° and *θ*_*i*_ = −10°, respectively, demonstrating the dual‐beam radiation capability.

**FIGURE 5:**
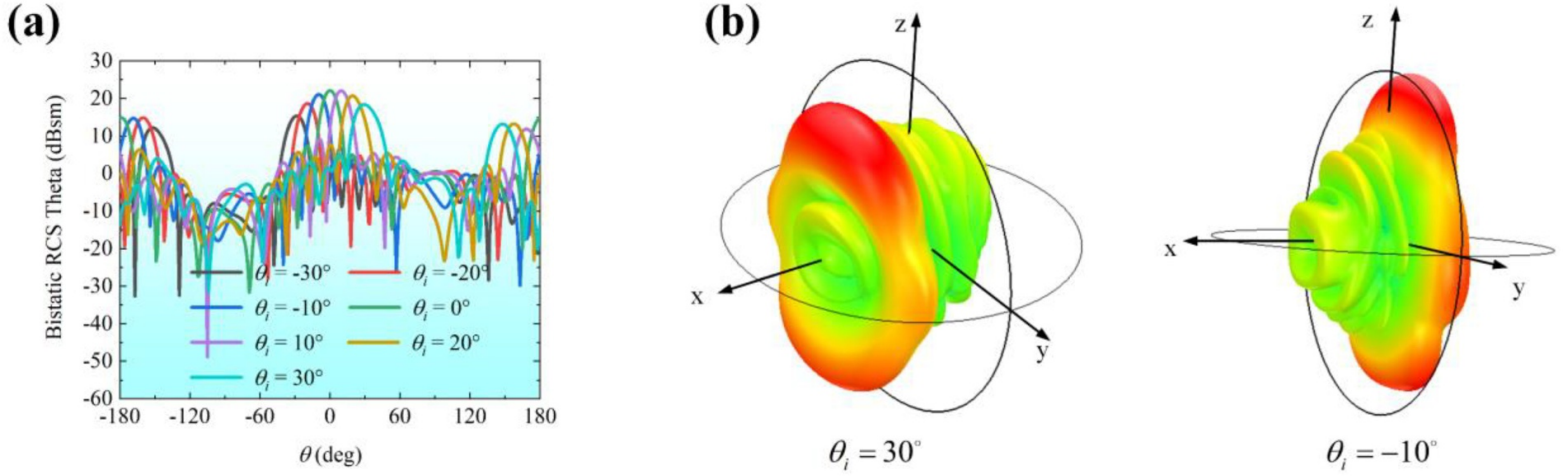
Simulation results of dual‐beam retrodirective array system at 10 GHz. (a) Bistatic RCS in *xoz* plane. (b) 3D bistatic RCSs in the direction of *θ*_*i*_ = 30° and *θ*_*i*_ = −10°.

The designed dual‐beam retrodirective array not only provides conventional retroreflection but also generates a secondary steerable beam. However, the direction of the secondary beam is not independently controllable—it varies with the angle of the incident wave, which limits its practical applicability. To overcome this constraint, a beam‐reconfigurable retrodirective array is further developed, as illustrated in Figure 6. A mechanically adjustable metal plate is positioned 40 mm beneath the transmitting array. When the plate is tilted to 45°, an incident wave from direction *θᵢ* in the *xoz* plane illuminates the array, prompting it to generate two beams. The retroreflective beam remains unchanged toward the source at (*θ* = *θᵢ*, *φ* = 0°). Meanwhile, the second beam, produced by the dual‐beam array, is reflected by the tilted metal plate into the *xoy* plane, toward the direction (*θ* = 90°, *φ* = 90°−*θᵢ*). When the metallic plate is rotated to *θ*_*m*_, the retroreflective beam at (*θ* = *θᵢ*, *φ* = 0°) and the steerable beam at (*θ* = 2*θ*_*m*_, *φ* = 90°−*θᵢ*) are simultaneously generated. This method provides dynamic control of the secondary steerable beam, which is completely independent of the retrodirective beam.

**FIGURE 6:**
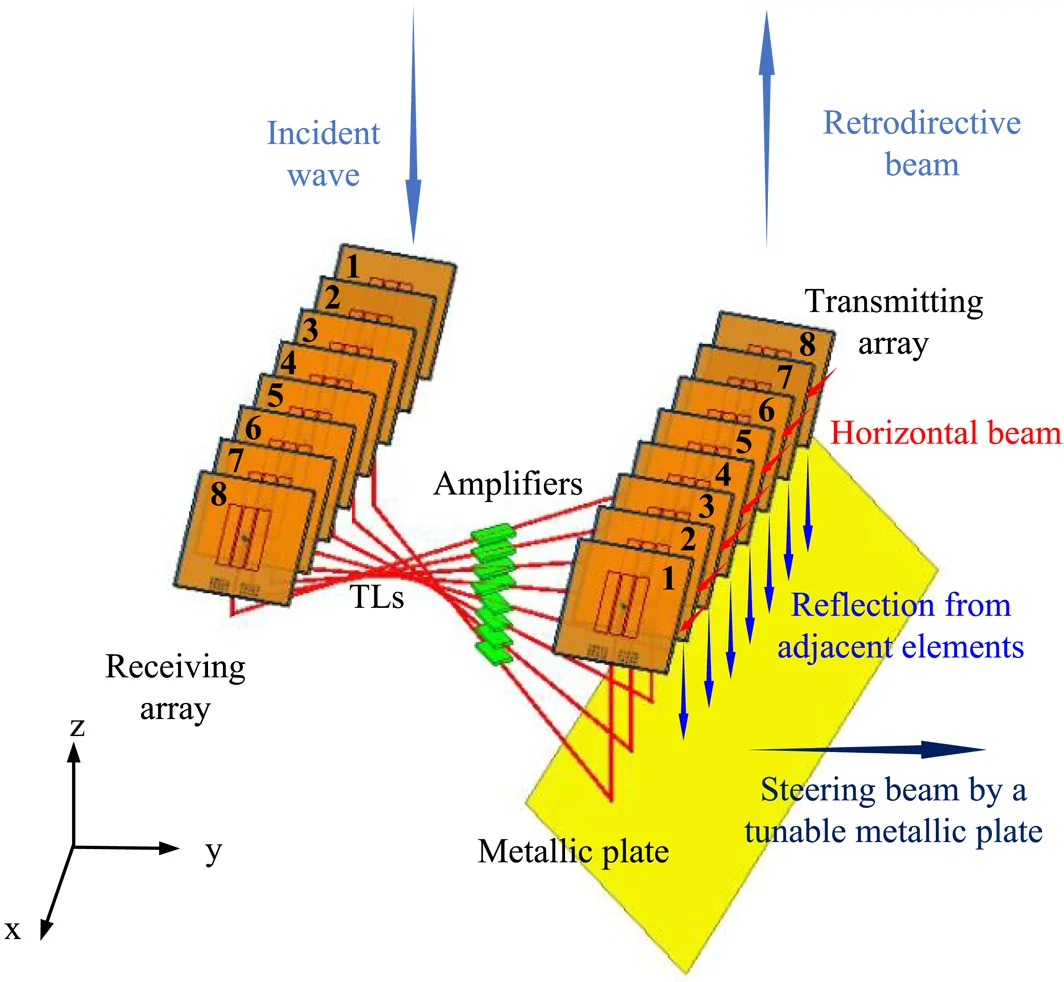
Beam‐reconfigurable retrodirective array system including the dual‐beam retrodirective array system and a metallic plate with a tunable tilt angle.

The proposed beam‐reconfigurable retrodirective array is simulated under the aforementioned parameters, with the metal plate tilted at 45° relative to the *z*‐axis. When illuminated by a wave incident at angles *θᵢ* ranging from −30° to +30° in the *xoz* plane, the array is expected to maintain its retroreflective beam within the same plane. Meanwhile, due to reflection by the metal plate, a secondary steerable beam emerges in the *xoy* plane, scanning from *φ* = 60° to *φ* = 120°. As shown in Figure 7a, the simulation confirms successful generation of a retroreflective beam in the *xoz* plane with good performance. Additionally, consistent with theoretical expectations, the steerable beam is also formed in the *xoy* plane, with its direction varying as a function of the incident angle *θᵢ*.

**FIGURE 7:**
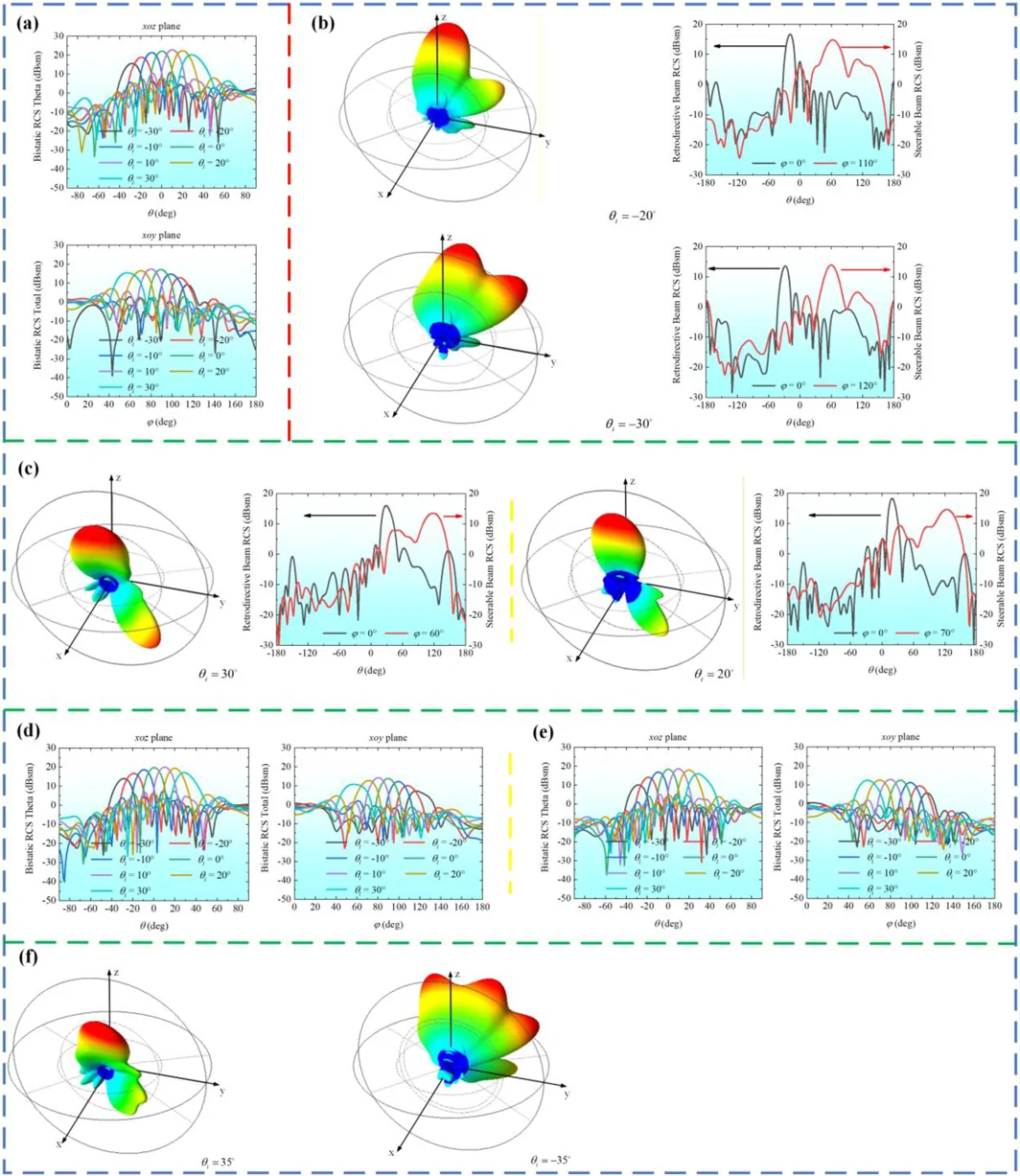
Simulation results of the beam‐reconfigurable retrodirective array system. (a) Bistatic RCSs in *xoz* and *xoy* planes for the metallic plate with a 45° tilt at 10 GHz. (b) Bistatic RCSs under the illumination at *θ*_*i*_ = −20° and *θ*_*i*_ = −30° for the metallic plate with a 30° tilt at 10 GHz. (c) Bistatic RCSs under the illumination at *θ*_*i*_ = 30° and *θ*_*i*_ = 20° for the metallic plate with a 60° tilt at 10 GHz. (d) Bistatic RCSs in *xoz* and *xoy* planes for the metallic plate with a 45° tilt at 9.8 GHz. (e) Bistatic RCSs in *xoz* and *xoy* planes for the metallic plate with a 45° tilt at 10.3 GHz. (f) Bistatic RCSs under the illumination at *θ*_*i*_ = −35° and *θ*_*i*_ = 35° for the metallic plate with a 45° tilt at 10 GHz.

To further explore the beam‐reconfigurable capability, the tilt angle of the metal plate is varied. Figure 7b,c show the corresponding scattering field patterns when the metal plate is tilted at 30° and 60° relative to the *z*‐axis, respectively. For a tilt angle of 30°, the array is illuminated by incident waves at *θᵢ* = −20° and *θᵢ* = −30° in the *xoz* plane. The corresponding steerable beams were observed at (*θ* = 60°, *φ* = 110°) and (*θ* = 60°, *φ* = 120°), along with the expected retroreflective beams at their respective incident directions. With the tilt angle increased to 60°, the array was illuminated by incident waves at *θᵢ* = 30° and 20° in *xoz* plane. In addition to the persistent retroreflective beams, the secondary beams were steered to (*θ* = 120°, *φ* = 60°) and (*θ* = 120°, *φ* = 70°). It should be noted that the scattering intensity of the steerable beam is lower than that of the retrodirective beam. This reduction arises from two factors. First, the horizontal beam is deflected by only seven elements, which induces partial energy loss. Second, unlike far‐field specular reflection, this reflection by the adjacent element occurs in the near field, leading to additional energy dissipation. Figure 7d,e illustrate the simulated bistatic RCS results at 9.8 and 10.3 GHz, respectively, which show similar variation trends to those at the center frequency of 10 GHz. This consistency demonstrates that the array maintains excellent retrodirective capability across the 9.8–10.3 frequency band. Additionally, Figure 7f presents the 3D scattering field patterns at 10 GHz under incident waves at the angles of +35° and −35°. It can be observed that the scattered pattern becomes distorted at excessively large incident angles, which is constrained by the inherent beamwidth of the array element (approximately 60°).

### 2.2 Experimental Verification

The array prototype was fabricated and assembled, and its RCS was measured in an anechoic chamber. As shown in Figure 8a, the prototype comprises two eight‐element linear arrays: the upper array serves as the receiving array, whereas the lower one acts as the transmitting array. A rotatable metal plate, positioned beneath the transmitting array, provides beam‐steering capability. All elements and the metal plate are mounted on a 3D‐printed fixture to ensure surface planarity. Eight PAs are placed behind the metal plate and connected to the transceiver elements via SMA‐to‐SMP RF cables to amplify the array signals.

**FIGURE 8:**
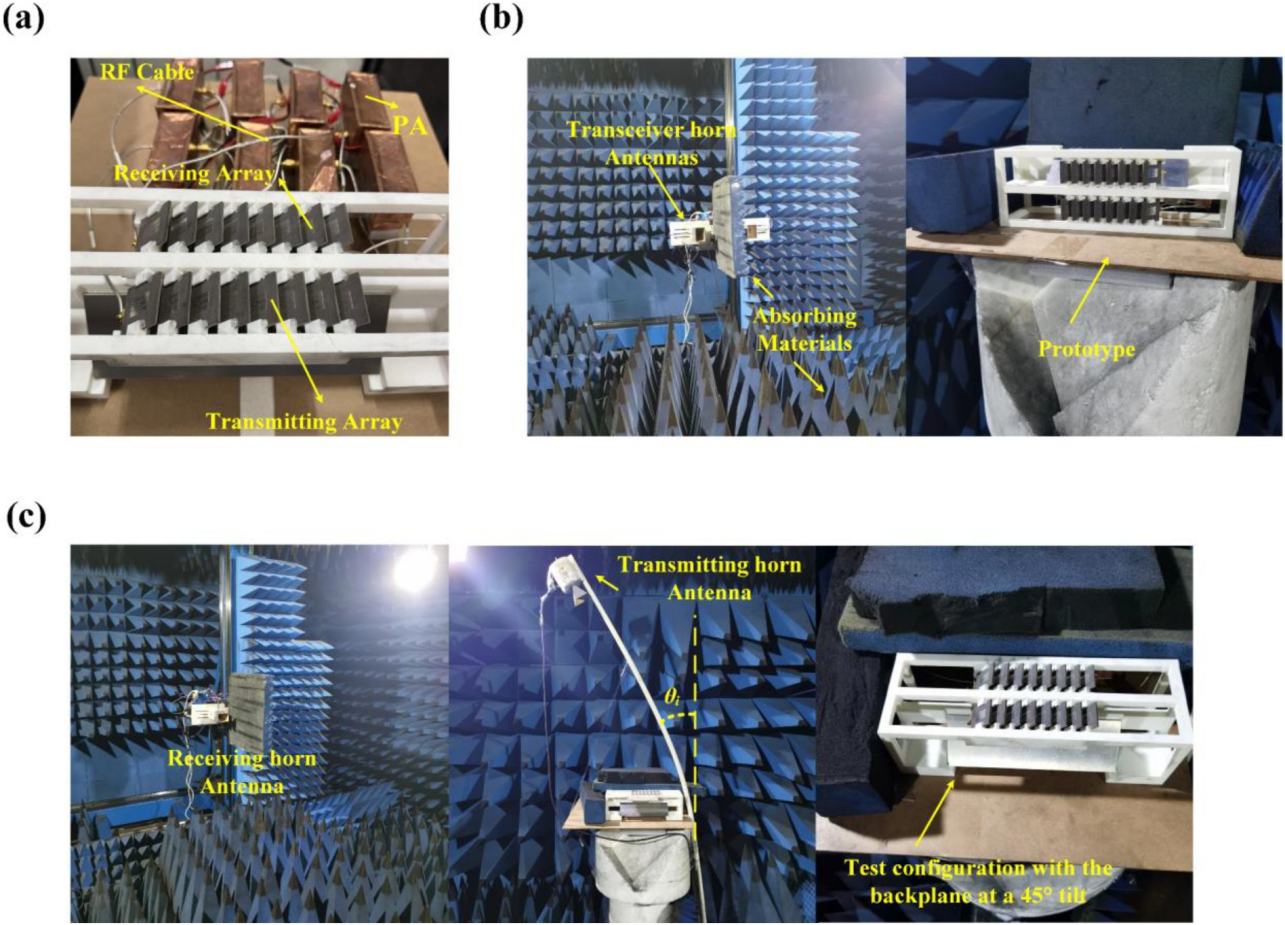
Prototype and measurement environment. (a) Prototype. (b) Measurement setup of the retrodirective beam. (c) Measurement setup of the steerable beam.

The prototype was evaluated in an anechoic chamber, with the test setups for the retrodiective beam and the steerable beam depicted in Figure 8b,c, respectively. In Figure 8b, two X‐band linearly polarized horn antennas (HD‐100SGAH10 from Hengda Microwave) were used as transmitting and receiving antennas, respectively. One horn was connected to the output port of an Agilent N5244A vector network analyzer (VNA), whereas the other was connected to the VNA's input port. Absorptive materials were placed between the two horns to minimize unwanted mutual coupling. The prototype was mounted on a low‐RCS foam turntable positioned in the far‐field region of the horn antennas, so as to reduce interference with the array's scattering field. A control system positioned outside the chamber was used to rotate the turntable, thus enabling monostatic scattering field measurements of the array across a range of incident angles.

The monostatic RCS measurement of the retrodirective beam was first conducted, with results shown in Figure 9a. The fabricated prototype exhibits excellent retroreflective performance, maintaining high scattering intensity within the ± 30° angular range. This confirms the array's ability to effectively form the first beam toward the incident direction. Figure 9b presents a comparison between simulated and measured monostatic RCS values. The simulated results in Figure 7a were adjusted downward by 3 dB to account for the total transmission path loss, comprising 1.5 dB insertion loss from each of the two RF cables, connecting every transceiver pair. The measured RCS of the array is lower than the simulated results, which can be attributed to fabrication tolerances of the radiation elements, as well as losses of the soldering variations in the RF connectors.

**FIGURE 9:**
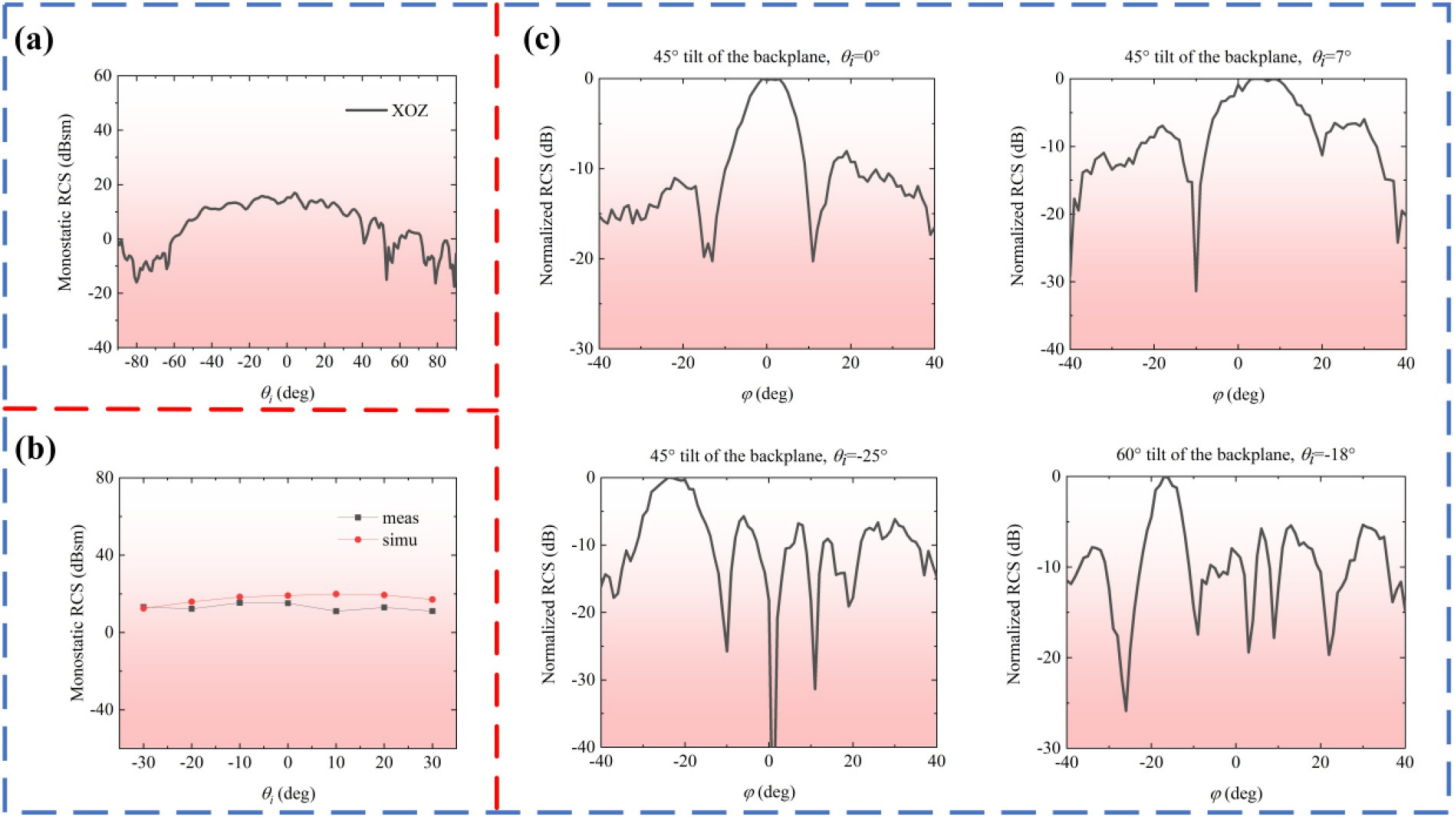
Measured RCS results. (a) Monostatic RCS for the retrodirective beam. (b) Comparison between simulated and measured monostatic RCSs for the retrodirective beam. (c) Measured results of the normalized scattering field for the steerable beam.

Figure 8c illustrates the measurement setup for characterizing the second steerable beam. Here, the array is vertically oriented. The transmitting horn is positioned above it, and the receiving horn is identical to that used in the previous measurement. Upon capturing the incident wave by the receiving array and reradiating it via the transmitting array, the resulting secondary beam is redirected into the horizontal plane by the metal plate. The transmitting horn rotates synchronously with the turntable to maintain a fixed illumination angle relative to the array. As the turntable rotates, the receiver horn, positioned in the far field within the horizontal plane, captures the reflected secondary beam, forming a bistatic scattering measurement.

Scattering field measurements were conducted for different tilt angles of metal plate (45° and 60°) and various incident wave directions. The results are summarized in Figure 9c. It can be observed that for both tilt angles, the metal plate consistently steers the secondary beam as expected under different incident angles, in good agreement with theoretical predictions. In addition, the secondary beam exhibits a wider beamwidth compared with the simulation results, which is caused by the array spacing errors introduced during fabrication and measurement. In conclusion, the proposed array not only maintains retrodirective reflection toward the incident direction but also generates a dynamically steerable scattering beam, thereby validating the operational principle and effectiveness of the designed beam‐reconfigurable retrodirective array.

## 3 Conclusion

This paper proposes a self‐designed dual‐beam microstrip element and develops an active retrodirective array system with beam reconfigurability. The radiating patch, featuring two etched slots and a coaxial feed, generates two independent beams in the X‐band, providing a physical basis for high‐performance retrodirection. At the system level, a mechanically tunable metal plate is innovatively placed beneath the transmitting array. This design retains the retroreflective function for incident waves while enabling dynamic steering of a secondary scattering beam to desired directions. Furthermore, by integrating multiple power amplifiers between the receiving and transmitting arrays, the system's RCS is significantly enhanced for operation in complex electromagnetic environments. Experimental results show that the system maintains stable retrodirective performance within a ± 30° angular range, with the secondary beam direction being flexibly controlled by adjusting both the metal plate's orientation and the incident wave direction. By enabling unique dual‐beam steering, this design overcomes the limitations of conventional retrodirective arrays and provides a feasible pathway for applications such as point‐to‐multipoint communication and wireless power transfer. Notably, compared with the electronic‐based reconfiguration, the proposed mechanical reconfiguration is simpler in implementation but suffers from low switching speed. Thus, the second beam steering is better suitable for the scenarios involving the moving objects at low to medium speeds. Further research will focus on methods to enhance its switching speed.

## Author Contributions

**Wen Yue Wei:** conceptualization, methodology, validation, data curation, writing – original draft, investigation. **Yan Shi:** conceptualization, methodology, writing – review and editing, supervision. **Li Jing Yang:** data curation, validation. **Jun Wei Wu:** writing – review and editing, methodology. **Qiang Feng:** writing – review and editing, validation. **Long Li:** writing – review and editing, supervision.

## Conflicts of Interest

The authors declare no conflicts of interest.

## Data Availability

The data that support the findings of this study are available from the corresponding author upon reasonable request.
